# Fluoxetine-Induced Hypoglycaemia in a Patient with Congenital Hyperinsulinism on Lanreotide Therapy

**DOI:** 10.4274/jcrpe.2818

**Published:** 2016-09-01

**Authors:** Dinesh Giri, Victoria Price, Zoe Yung, Mohammed Didi, Senthil Senniappan

**Affiliations:** 1 Alder Hey Children’s Hospital, Clinic of Pediatric Endocrinology, Liverpool, United Kingdom

**Keywords:** Fluoxetine, selective serotonin reuptake inhibitor, hypoglycaemia, Hyperinsulinism

## Abstract

Antidepressant drugs are reported to cause alterations in blood glucose homeostasis in adults with diabetes mellitus. We report a patient with persistent congenital hyperinsulinism (CHI) who developed recurrent hypoglycaemia following fluoxetine therapy. This 15-year-old girl was initially managed with diazoxide therapy. She developed troublesome hypertrichosis, which affected her quality of life adversely. Diazoxide was then slowly weaned and stopped with the introduction of octreotide, to which she responded well. Subcutaneous lanreotide (long-acting somatostatin analogue) was subsequently commenced (30 mg, once monthly) as injecting octreotide multiple times a day was proving to be difficult for the patient. The continuous blood glucose monitoring on monthly lanreotide injections revealed good glycaemic control. Six months later, she developed depression due to psychosocial problems at school. She was started on fluoxetine by the psychiatry team. She subsequently developed recurrent symptomatic hypoglycaemic episodes (blood glucose <3.5 mmol/L) and fluoxetine was discontinued, following which the hypoglycaemic episodes resolved within a week. Fluoxetine has been associated with hypoglycaemia in patients with diabetes mellitus. We report, for the first time, hypoglycaemia secondary to fluoxetine in a patient with CHI.

**WHAT IS ALREADY KNOWN ON THIS TOPIC?**

Diazoxide is used as the first-line medication in congenital hyperinsulinism (CHI). Troublesome hypertrichosis may occur as a side-effect which may lead to depression, particularly in adolescent females. Fluoxetine, an antidepressant, has been implicated to cause disturbances in glucose homeostasis in patients with diabetes.

**WHAT THIS STUDY ADDS?**

Fluoxetine can cause or worsen hypoglycaemia in patients with CHI. This has not been reported in the literature before.

## INTRODUCTION

Congenital hyperinsulinism (CHI) is a disorder caused by dysregulated insulin secretion from the beta cells of the pancreas and is the major cause of hypoglycaemia during infancy and childhood (1). Antidepressant agents have been implicated to contribute to glucose dysregulation, causing both hypo- and hyperglycemia, depending on the specific medication used (2). Such disturbances have been reported in normal subjects as well as in patients with diabetes mellitus (DM). Selective serotonin reuptake inhibitors (SSRIs), in particular fluoxetine, have been associated with hypoglycaemia, reduced awareness of hypoglycaemic episodes, and increased insulin sensitivity in patients with DM (3,4,5,6,7). It is well known that unrecognised and untreated hypoglycaemia can be devastating and can potentially cause neurological damage. Moreover, the hypoglycaemic episodes occurring as a side-effect of these medications can have a negative psychological impact in a chronic condition such as DM and persistent forms of CHI. It is therefore important to be aware of the potential impact of antidepressants on glycaemic control in patients with DM and CHI. Fluoxetine-induced hypoglycaemia has been reported in the literature in adult patients with DM with paucity of literature in the paediatric population. Also, to our knowledge, there are no previous published report of fluoxetine-induced hypoglycaemia in patients with CHI. We report a 15-year-old girl with CHI who developed persistent and recurrent hypoglycaemia secondary to fluoxetine.

## CASE REPORT

A 15-year-old girl with a diagnosis of CHI was referred to our clinic from a District General Hospital. She was born to healthy non-consanguineous Caucasian parents. She was noted to be hypoglycemic since birth and subsequent diagnostic work up for hypoglycaemia led to the diagnosis of CHI. CHI was defined as an inappropriately elevated insulin level (100 pmol/L) during hypoglycaemia (2 mmol/L) with suppressed fatty acids (<100 µmol/L) and 3-hydroxy butyrate (<100 µmol/L). Genetic analysis of the patient revealed a de novo heterozygous ABCC8 mutation. She was started on diazoxide (5 mg/kg/day), in conjunction with chlorothiazide (7 mg/kg/day), to which she responded well. Chlorothiazide was subsequently weaned and stopped when the patient was 5 years of age. At this time, 18-Fluro DOPA positron emission tomography (PET) computed tomography scan of the pancreas revealed diffuse disease. While glycemic control was optimal on diazoxide, its long-term use caused hypertrichosis, a well-known side effect of the drug. The troublesome hypertrichosis continued through her teenage years and had a significant impact on her quality of life. She suffered from depression and was missing a lot of school. She was referred for therapies including wax therapy and laser to help with her hirsutism. Unfortunately, the hypertrichosis and hirsutism were not amenable to these therapies. This was imposing a negative impact on her quality of life with her committing deliberate self-harm on a few occasions. The patient was in continuous need for psychological assistance and support. She was electively admitted to our pediatric inpatient unit to decide on an alternative medical treatment for CHI. A trial off diazoxide was performed whereby, diazoxide was gradually weaned and stopped for 72 hours. However, this led to subsequent development of recurrent symptomatic hypoglycaemia (blood glucose <3.5 mmol/L). Further investigations revealed a persistent hyperinsulinaemic hypoglycaemia with a plasma insulin concentration of 96 pmol/L and a suppressed plasma ketone level (free fatty acids-608 µmol/L, 3 hydroxy butyrate-47 µmol/L) when the plasma blood glucose was 2.6 mmol/L. Octreotide (a somatostatin analogue) was then commenced as four times daily subcutaneous injection. Baseline investigations including ultrasound of gall bladder, thyroid function tests, and insulin-like growth factor-1 (IGF1) level prior to commencement of octreotide were within normal limits. Octreotide was started at 5 mcg/kg/day and was gradually built up to 15 mcg/kg/day as 4 divided subcutaneous injections. A good glycemic response was noted at this dose and she was able to tolerate a fast for a period of 24 hours with no evidence of hypoglycaemia. However, a 4 times daily injection therapy was becoming too labour intense for her and she did not tolerate this intense therapy. Hence, lanreotide (long-acting somatostatin analogue) was commenced as a subcutaneous injection 30 mg once monthly and octreotide was gradually weaned and stopped. The continuous blood glucose monitoring system following the administration of lanreotide revealed good glycaemic control with no episodes of hypoglycaemia. She also noted significant improvement in her quality of life and her school attendance improved.

**Figure 1:**
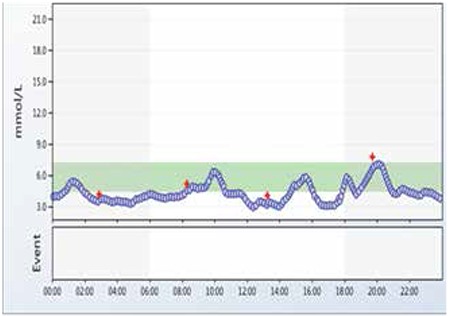
Continuous blood glucose monitoring on fluoxetine

**Figure 2:**
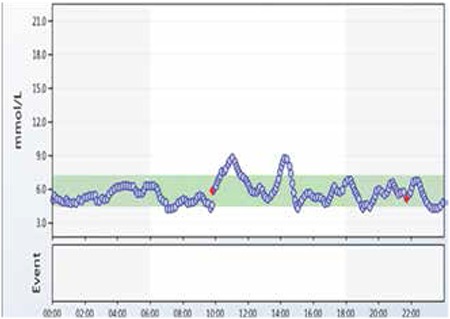
Continuous blood glucose monitoring off fluoxetine

Six months later, she developed depression due to psychosocial problems at school. She was assessed by a psychiatrist and was commenced on fluoxetine at a dose of 20 mg once daily. A week later, after the commencement of fluoxetine, the patient developed recurrent symptomatic hypoglycaemic episodes (blood glucose <3.5 mmol/L). The blood test during one of these hypoglycaemic episodes showed suppressed plasma insulin (<14 pmol/L) and c-peptide (<100 pmol/L) concentrations. Continuous blood glucose monitoring (CGM) was performed while the patient was on fluoxetine. The CGM did reveal multiple hypoglycaemic episodes (blood glucose <3.5 mmol/L) (Figure 1). After discussing with the psychiatric team, fluoxetine was discontinued, following which the hypoglycaemic episodes resolved within a week. CGM, two weeks after the discontinuation of fluoxetine, revealed a resolution of the hypoglycaemic episodes (Figure 2). The patient’s depressive symptoms slowly improved over time with the help of counselling.

## DISCUSSION

Persistent CHI and DM are chronic medical conditions that cause disturbances in glucose homeostasis. DM is one of the commonest chronic diseases in childhood. Hypoglycaemia is a common occurrence in both these conditions. Moreover, chronic illnesses usually carry the risk of associated depression. For instance, in adults with DM, the risk of co-morbid depression is 8.5% to 20.0% higher than the general population (8). Treatment of depression associated with chronic illness is important as delay or non-treatment may lead to poor glycaemic control. For instance, in patients with DM, depression is associated with hyperglycaemia and an increased risk for diabetic complications and treatment of depression is associated with improved glycaemic control (9). In our patient, the extent of psychosocial problems at her school was affecting her adversely and warranted the need for psychiatric counselling and antidepressant therapy.

SSRIs are one of the most commonly used antidepressant medications and are usually the first choice for depression because of fewer side effects than most other types of antidepressants (10). Fluoxetine, an SSRI, is an antidepressant that leads to enhanced serotonergic neurotransmission. Fluoxetine blocks serotonin reuptake and increases serotonin stimulation of receptors during the acute phase, and chronic use leads to desensitization of somatodendritic 5-HT1A and of terminal autoreceptors with an overall clinical effect of increased mood and decreased anxiety (11). Fluoxetine is indicated for use in pediatric patients over 8 years of age with multiple mental health diagnoses, including depression. It is a daily oral tablet, which can be titrated according to effect (12).

In our patient, diazoxide was providing optimal management for hypoglycaemia but due to significant hypertrichosis interfering with lifestyle and not amenable to therapies, octreotide was introduced which proved to be effective. As octreotide involves multiple injections which were proving to be difficult for the patient, lanreotide (long-acting somatostatin analogue, 30 mg as subcutaneous injection, once a month) was introduced and was providing optimal glycaemic control (13), until our patient developed hypoglycaemic episodes following fluoxetine therapy.

Antidepressant drugs, particularly SSRIs, are reported to have a variety of effects on glucose homeostasis. SSRIs may cause hypoglycaemic unawareness secondary to autonomic dysfunction (14). Various mechanisms have been hypothesised to explain fluoxetine-induced hypoglycaemia. PET in healthy subjects has shown a decrease in the relative cerebral glucose metabolism in the amygdaloid complex, hippocampal formation, and ventral striatum with fluoxetine when compared to a placebo (15). McIntyre et al (2) suggested a central mechanism of action of fluoxetine. Potter van Loon et al (7) have shown that fluoxetine improves peripheral and hepatic insulin action in obese insulin-resistant subjects irrespective of its weight lowering effect. In a randomized, double-blind, placebo-controlled trial, insulin-mediated glucose disposal was measured in 12 obese patients with non-insulin dependent DM (NIDDM) on diet alone before and after four weeks of treatment with either placebo or fluoxetine and it was found that fluoxetine improves insulin-mediated glucose disposal in obese patients with NIDDM (6). These studies suggest that fluoxetine may have a role in increasing the insulin sensitivity. In our patient with CHI, a condition that causes an excessive and dysregulated insulin secretion, we believe that the introduction of fluoxetine has caused an increase in insulin sensitivity thereby resulting in recurrent hypoglycaemic episodes. Resolution of the hypoglycaemic episodes two weeks after stopping of fluoxetine substantiates this hypothesis.

In conclusion, this case demonstrates that hypertrichosis, a common side effect of diazoxide therapy for CHI, can result in depression, particularly in adolescent females. Management of diazoxide-responsive patients using long-acting somatostatin analogue, lanreotide, might be beneficial both in terms of increasing the patient’s compliance and avoiding the side effects which usually constitutes the main concern for families and patients. Although fluoxetine-related hypoglycaemia has been reported in patients with DM, it may not be a widely recognized phenomenon among many health professionals. We report, for the first time, hypoglycaemia secondary to fluoxetine in a patient with CHI. We suggest that close blood glucose monitoring should be undertaken in patients with disorders of glucose homeostasis who are commenced on antidepressant therapy.

## Ethics

Informed Consent: It was taken.

Peer-review: Externally peer-reviewed.
